# Anaemia in Pregnancy: Prevalence, Risk Factors, and Adverse Perinatal Outcomes in Northern Tanzania

**DOI:** 10.1155/2018/1846280

**Published:** 2018-05-02

**Authors:** Grace Stephen, Melina Mgongo, Tamara Hussein Hashim, Johnson Katanga, Babill Stray-Pedersen, Sia Emmanueli Msuya

**Affiliations:** ^1^Institute of Public Health, Department of Community Health, Kilimanjaro Christian Medical University College (KMUCO), P.O. Box 2240, Moshi, Tanzania; ^2^Better Health for African Mothers and Children (BHAMC) Project, P.O. Box 8418, Moshi, Tanzania; ^3^Institute of Clinical Medicine, Faculty of Medicine, University of Oslo, Oslo, Norway; ^4^Institute of Basic Medical Sciences, Faculty of Medicine, University of Oslo, Oslo, Norway; ^5^Ocean Road Cancer Institute, Directorate of Cancer Prevention Services, P.O. Box 3592, Dar es Salaam, Tanzania; ^6^Division of Gynaecology and Obstetrics, Oslo University Hospital, Rikshospitalet, 0863 Oslo, Norway; ^7^Department of Community Medicine, Kilimanjaro Christian Medical Centre (KCMC), Moshi, Tanzania

## Abstract

**Background and Objective:**

Anaemia in pregnancy is a public health problem in developing countries. This study aimed to determine the prevalence, risk factors, and adverse perinatal outcomes of anaemia among pregnant women in Moshi Municipal, Northern Tanzania.

**Methods:**

This was a follow-up study conducted from October 2013 to June 2015. A total of 539 pregnant women were enrolled in this study. Interviews were conducted followed by determination of haemoglobin level. Women were followed up at delivery and at 7 days and 28 days after delivery.

**Results:**

A total of 529 women were included in this analysis. Their mean age was 25.8 (SD 5.73). The prevalence of anaemia was 18.0% and 2% had severe anaemia. The clinic of recruitment and low education level of the women were the factors that were independently associated with anaemia during pregnancy. At delivery, there were 10 stillbirths, 16 low birth weight (LBW) newborns, and 2 preterm birth cases. No association was found between anaemia and LBW, preterm birth, or stillbirths.

**Conclusion:**

Anaemia in pregnancy was a mild public health problem in the study setting of Northern Tanzania.

## 1. Introduction

Anaemia during pregnancy is a public health problem especially in developing countries and is associated with adverse outcomes in pregnancy [1]. World Health Organization (WHO) has defined anaemia in pregnancy as the haemoglobin (Hb) concentration of less than 11 g/dl [2]. According to WHO, anaemia is considered to be of a public health significance or problem if population studies find the anaemia prevalence of 5.0% or higher. Prevalence of anaemia of ≥40% in a population is classified as a severe public health problem [3].

Global data shows that 56% of pregnant women in low and middle income countries (LMIC) have anaemia [1]. The prevalence of anaemia is highest among pregnant women in Sub-Saharan Africa (SSA) (57%), followed by pregnant women in Southeast Asia (48%), and lowest prevalence (24.1%) was found among pregnant women in South America [3]. Tanzania Demographic and Health Surveys reported a slight decrease in the prevalence of anaemia among pregnant women from 58% in 2004/05 to 53% in 2010 [4, 5]. Other studies conducted in Tanzania have reported a higher prevalence of anaemia among pregnant women: 68% in Dar es Salaam and 47% in Moshi [6, 7].

The causes of anaemia during pregnancy in developing countries are multifactorial; these include micronutrient deficiencies of iron, folate, and vitamins A and B12 and anaemia due to parasitic infections such as malaria and hookworm or chronic infections like TB and HIV [7–11]. Contributions of each of the factors that cause anaemia during pregnancy vary due to geographical location, dietary practice, and season. But in Sub-Saharan Africa inadequate intake of diets rich in iron is reported as the leading cause of anaemia among pregnant women [10, 11].

Anaemia during pregnancy is reported to have negative maternal and child health effect and increase the risk of maternal and perinatal mortality [12, 13]. The negative health effects for the mother include fatigue, poor work capacity, impaired immune function, increased risk of cardiac diseases, and mortality [1, 13, 14]. Some studies have shown that anaemia during pregnancy contributes to 23% of indirect causes of maternal deaths in developing countries [1].

Anaemia in pregnancy is associated with increased risk of preterm birth and low birth weight babies [1, 6, 7, 15]. Preterm and LBW are still the leading causes of neonatal deaths in developing countries like Tanzania contributing to 30% of the deaths [16]. It has also been associated with increased risk of intrauterine deaths (IUFD), low APGAR score at 5 minutes, and intrauterine growth restriction (IUGR) which is a risk for stunting among children of less than two years [6, 7, 17].

Tanzania as a country has strengthened different interventions to reduce the burden of anaemia during pregnancy. The interventions during pregnancy include anaemia screening during pregnancy and treatment, giving a combination of folic acid (FeFo) and iron supplements for three months, deworming, intermittent prophylaxis treatment for malaria (IPTp) with sulfadoxine pyrimethamine (SP) from 14 weeks, free provision of mosquito treated nets, and health education during the antenatal visits [18]. Few studies have evaluated the burden of anaemia and its effect in pregnant outcomes in Tanzania after scaling up of preventive interventions. Data for studies by Kidanto et al. [6] and Msuya et al. [7] that have shown prevalence of anaemia in pregnancy in Dar es Salaam and Moshi Tanzania as well as TDHS of 2010 were collected between 2004 and 2010, before strengthening interventions targeting anaemia in pregnancy and interventions improving overall maternal and neonatal health. There is a need of having current information on burden and effects of anaemia during pregnancy after these multiple interventions. Therefore, this study aims to determine prevalence, risk factors, and associated perinatal adverse perinatal outcomes of anaemia during pregnancy in Moshi Municipality.

## 2. Methods

### 2.1. Study Design and Study Setting

The study was part of larger cohort study that aimed to describe the effects of Sexually Transmitted Infections/Reproductive Tract Infections and anaemia on pregnancy outcomes and child growth in Moshi Municipality, Tanzania [19]. The study was conducted between October 2013 and June 2015 in two health care centres, that is, Majengo and Pasua health centres in Moshi Municipality. The two clinics are the largest primary health centres in Moshi Municipality.

The larger study enrolled women in their third trimester of pregnancy and followed them at birth, at 7 days postdelivery, and monthly up to 9 months and every postdelivery. Enrolment of pregnant women was conducted in October 2013 to April 2014 and follow-up of mothers and their infants up to 9 months was completed in June 2015 [20]. This paper used data that was collected from enrolment up to seven days postdelivery.

Moshi Municipality has a population of 184,292 and 56,848 women of reproductive age [21]. The total deliveries in Moshi Municipality in 2017 was 12040. There are 4 hospitals, 8 health centres, and 32 dispensaries where 25 health facilities provide reproductive and child health services. The study was conducted at 2 government health centres, Majengo and Pasua, which include a huge population of pregnant woman in Moshi urban area and have the capacity to deliver about 1301 and 955 women per year, respectively. The two clinics provide services to approximately 3600 and 3000 pregnant women in Majengo and Pasua, respectively. In 2017 Majengo had 1343 deliveries and Pasua had 1001 deliveries.

### 2.2. Sample Size Calculations

Sample size was estimated by using the following formula. The minimum sample that was required for this study was 390 pregnant women.

(1) $$\begin{array}{c} N=\frac{Z^{2}*P\left(1-P\right)}{\varepsilon^{2}}, \end{array}$$

where *N* is estimated minimum sample size; *Z* is confidence level at 95% (standard value is 1.96); *P* is proportion (prevalence of anaemia during pregnancy 53% TDHS, 2010); *ɛ* is precision at 95% CI = 0.05.

(2) $$\begin{array}{c} N=\frac{{\left(1.96\right)}^{2}\times 0.53\left(1-0.53\right)}{{\left(0.05\right)}^{2}}. \end{array}$$

### 2.3. Study Population and Procedures

The study population included all pregnant women who were in their third trimester and attending for routine care at the two primary health care clinics between October 2013 and April 2014. The study excluded women who reported they will relocate/move after delivery and those who did not consent.

Women were informed about the study aims and follow-up schedule and those agreeing to participate gave a signed consent. Face-to-face interviews using questionnaire were conducted by trained research assistants who were nurses/doctors and underwent one-week training. The interviews were conducted in Swahili language. The information collected included social demographic characteristics, economic characteristics, reproductive health history, feeding practices, and intended place of delivery. After the interviews, clinical examinations were conducted and blood sample was collected for diagnosis of HIV, STIs, and haemoglobin levels [19].

**Figure 1:**
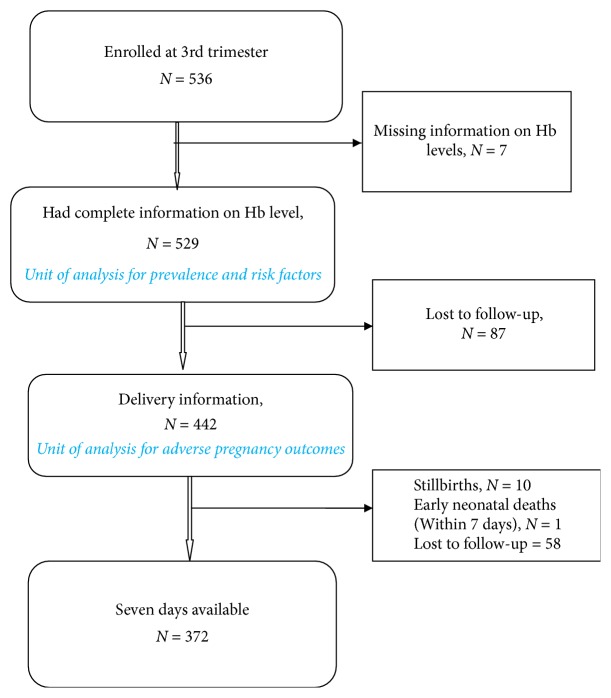
Follow-up of pregnant women up to 7 days.

A total of 536 pregnant women were enrolled, but analysis was done on 529 women who had complete information of haemoglobin levels; see Figure 1.

### 2.4. Data Processing, Categorization, and Analysis

The data were entered, cleaned, and analysed by using SPSS version 20. Descriptive statistics was used to summarize data. Proportions were used for categorical variables and mean or median with respective measures of dispersion for numerical variables. The Odds Ratio (OR) with 95% Confidence Interval (CI) was used to measure the strength of association between anaemia and exposure variables (sociodemographic, economic, nutrition, and reproductive health characteristics) as well as association between anaemia and adverse pregnancy outcomes (LBW, preterm, and stillbirth). Logistic regression analysis was performed to control for the confounders. The *p* value of less than 0.05 was considered as a statistically significant result.

*Categorization of Variables.* A pregnant woman was considered anaemic if haemoglobin was <11 g/dl [2]. Severity of anaemia was measured as follows: mild if Hb was 9.0–10.9 g/dl; moderate if Hb was 7.0–8.9 g/dl; and severe if Hb was <7.0 g/dl [2]. Age of participants which was collected as numerical variable was categorized (14–24, 25–34, and 35–49), as well as income per month (<60,000 Tsh, 60,000–200,000 Tsh, and >200,001 Tsh), partners age (15–24, 25–34, and 35+), being gravida (first, second, third, or more pregnancies), parity (1, 2, 3, 4, and 5+), frequency of antenatal care visits (1, 2-3, and 4+), pregnancy interval (≤24 months and >24 months), gestation age at delivery (<37 and ≥37), and number of meals per day (1, 2, or 3 or more meals per day). Preterm delivery was categorized as <37 weeks of gestation age, low birth weight was categorized as < 2500 grams, and early neonatal death is the death during the first 7 days of life [22].

### 2.5. Ethical Consideration

The permission to conduct this study was sought from Kilimanjaro Christian Medical University College committee. The study was granted ethical clearance certificate number 945. Participants who were enrolled gave a signed consent. Participants who were found to have anaemia received free treatment and counselling according to Tanzania National treatment guidelines. Numbers instead of names were used in all the questionnaires and laboratory forms.

## 3. Results

### 3.1. Demographic and Reproductive Health Characteristics of the Women

The age of the 529 participants ranged from 15 to 46 years with mean age of 25.8 (SD 5.73) years. Majority of the participants were married/cohabiting, 479 (89%) and unemployed, 209 (61.1%), and 355 (67.9%) had an income per month less than 60,000 Tanzanian shillings (Tsh).

For majority of the 529 women (96.7%) this was their second or third antenatal visit, and 88% reported they have received iron supplementation during current pregnancy, Table 1. Most of the women (89%) reported an interpregnancy interval of ≥24 months. Other demographic and reproductive health characteristics are shown in Table 1.

### 3.2. Prevalence of Anaemia among Pregnant Women

**Figure 2:**
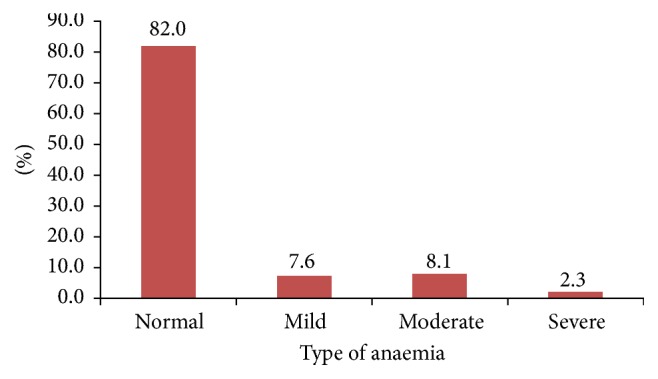
Severity of anaemia in pregnancy among women in Moshi Municipality.

The prevalence of anaemia was 18.0% (*n* = 95). Forty women had mild, 43 moderate, and 12 severe anaemia, Figure 2.

### 3.3. Factors Associated with Anaemia in Pregnancy

Table 2 shows association between anaemia and several predictor variables. Women who were recruited from Pasua clinic had two times higher odds being anaemic (OR; 2.1; 95% CI 1.3–3.3) compared to women who were recruited from Majengo health centre. Women with secondary education or higher had 76% less odds of having anaemia compared to others. Other factors like age, marital status, occupation, income, and alcohol intake were assessed but were not associated with anaemia during pregnancy.

Women who attended ANC 4 or more times had lower prevalence of anaemia (17.4%) than those who attended only once (35.3%); women who reported having received iron supplementation in current pregnancy had lower prevalence (20.2%) than those who have not received any supplementation (29.5%), but the difference was not statistically significant. Other factors that were analysed but were not associated with anaemia during pregnancy include gravida, parity, history of miscarriage, pica habits, HIV status, and gestational age.

Food security or household characteristics (water source for sanitation, owning toilet facility, household ownership, land ownership, history of food insecurity, number of meals taken per day, and intake of meat or fish) were assessed but none was associated with anaemia in pregnancy.

Table 3 shows the results of logistic regression analysis. Education and clinic of enrolment remained significantly associated with anaemia in pregnancy. Women enrolled at Pasua health centre had twice the odds of being anaemic compared to women from Majengo. Women with primary and secondary education or more had 72% and 79% significantly less odds of having anaemia compared to women with no formal education.

### 3.4. Birth Outcomes among Women in Moshi Municipality

Among 529 pregnant women who had complete information on Hb, 83.6% (*n* = 442) had delivery information, Figure 1. There were no difference in anaemic status between those women who had information at delivery and those who did not have information at delivery (*p* = 0.849).

At delivery, there were 10 stillbirths (2.3%), 16 low birth weight newborns (3.6%), and 2 (0.45%) preterm birth cases. Two out of 432 infants died within the first 7 days (0.5%).

No association was found between anaemia and low birth weight, preterm birth, or stillbirths in Moshi Municipality, Table 4.

## 4. Discussion

The study findings showed that prevalence of anaemia during pregnancy from the two selected health centres in Moshi Municipal was 18.0%. The clinic of recruitment and secondary or higher education among women were factors that were associated with anaemia in pregnancy. Anaemia in pregnancy was not associated with adverse pregnancy outcomes in this setting.

The prevalence of anaemia in the selected two clinics was lower compared to 47.4% as reported by Msuya and colleagues who collected their data about twelve years ago [7]. This may imply an improvement in maternal nutrition in this setting as well as general health and care during pregnancy. Over the years, the government has strengthened the antenatal care (ANC) services and every pregnant woman is given iron supplementation to combat anaemia, deworming, malaria prophylaxis, and mosquito nets [23]. Nowadays pregnant women have to take malaria prophylaxis and deworms in front of the health care provider. This increases the uptake of medication and hence prevents anaemia that can be caused by mosquitoes or helminthes. The prevalence of anaemia during pregnancy has been reported by other researchers to range from 32% to 62.2% [6, 7, 24].

Women who had secondary or higher education were less likely to be anaemic compared to their counterparts. Education has been reported to reduce the risk of being anaemic in several studies. Educated pregnant women have better income and eat nutritious food and hence do not get nutritional anaemia [5]. A study in Ethiopia also reported higher prevalence of anaemia among pregnant women who had no education [25]. Secondary and higher education had been associated with several other good maternal and child outcomes like higher frequency of exclusive breastfeeding, attending for antenatal care visits for 4 or more recommended visits, utilization of skilled attendance during delivery, and health care seeking when the children have pneumonia or malaria [5]. Women education and empowerment are not within health sector and there is a need for multisectoral collaboration in combating anaemia and other maternal health problems.

This study shows that women who were recruited from Pasua clinic were two times more likely to be anaemic than women who were recruited from Majengo clinic. Previous studies have shown that women living in Pasua have poor living standards and low income of less than one dollar per day compared to women at Majengo [7]. Poor income leads to limited access to nutritious diets and is associated with poor eating habits that might lead to anaemia. A study in Ethiopia showed that women with low income were more anaemic than women with higher income [26, 27].

In this study there was low occurrence of negative pregnancy outcomes: LBW (3.6%), preterm births (0.5%), and stillbirth (2.3%). The occurrence of negative birth outcomes was low compared to national prevalence of 13% for LBW and 12.7% for preterm birth [28]. This is contrary to findings by other researchers in Dar es Salaam and Moshi, Tanzania [6, 7], and researchers in Ethiopia, India, and Pakistan [15, 29, 30].

*Strength and Weakness of the Study*. Diagnosis of anaemia was based on laboratory analysis and did not depend on clinical assessment as reported by other researchers. Information on birth outcomes for women who were lost to follow-up from enrolment to delivery might have affected the prevalence of birth outcomes. It may be that those who were lost to follow-up experienced negative pregnancy outcomes and did not see the importance of returning for follow-up, hence underestimating occurrence of pregnancy outcomes. The other causes of negative birth outcomes like diabetes and preeclampsia were not assessed.

## 5. Conclusion

Anaemia in pregnancy was a mild public health problem in Northern Tanzania. The main risk factors were found to be the place of residence and education level of the pregnant woman. Ongoing interventions to target anaemia during pregnancy seem to be working in this setting and they should reach universal coverage. Further, we recommend ongoing education about effects of anaemia especially among women with low education and population of adolescent women and women of reproductive age in general.

## Figures and Tables

**Table 1:** Sociodemographic and reproductive health characteristics of the pregnant women (*N* = 529).

Variable name	Number (%)
*Mothers characteristics*	
Age (years)	
14–24	259 (49.0)
25–34	224 (42.3)
35–49	46 (8.7)
Level of education	
None	12 (2.3)
Primary	320 (60.5)
Secondary or higher	197 (37.2)
Marital status	
Married/cohabiting	479 (89.4)
Single/widow/divorced	56 (10.4)
Occupation (*N* = 327)^*∗*^	
Unemployed	209 (61.1)
Employed	30 (8.8)
Businesswomen	103 (30.1)
Income category for women (*N* = 523)^*∗*^	
<60,000 Tsh	355 (67.9)
60,000–200,000 Tsh	143 (27.3)
>200,000 Tsh	25 (4.8)
Alcohol intake	
No	457 (86.4)
Yes	72 (13.6)
Gravida	
First pregnancy	186 (35.2)
Second pregnancy	170 (32.1)
Third pregnancy and above	173 (32.7)
Interpregnancy interval (*N* = 301)	
<24 months	32 (10.6)
≥24 months	269 (89.4)
Antenatal care visit current (*N* = 517)	
1 visit	17 (3.3)
2-3 visits	339 (65.6)
4+ visits	161 (31.1)
Have received iron supplementation at current pregnancy (*N* = 356)^*∗*^	
No	44 (12.4)
Yes	312 (87.6)
Pica habits during this pregnancy (*N* = 363)^*∗*^	
No	138 (38.0)
Yes	225 (62.0)
HIV status at current pregnancy (*N* = 528)^*∗*^	
Negative	496 (93.8)
Positive	32 (6.1)

^*∗*^Variables with missing information.

**Table 2:** Sociodemographic, nutrition, and reproductive characteristics factors associated with anaemia in pregnancy (*N* = 529).

Variable	*N*	Anaemia (Hb < 11 g/dl) *n* (%)	Unadjusted OR (95% CI)	*p* value
Name of clinic enrolled				
Majengo HC	235	29 (12.3)	1	
Pasua HC	294	66 (22.4)	2.1 (1.28–3.31)	0.003
Age (years)				
14–24	259	48 (18.5)	1	
25–34	224	43 (19.2)	1.04 (0.66–1.65)	0.852
35–49	46	4 (8.7)	0.42 (0.14–1.22)	0.112
Level of education				
None	12	5 (41.7)	1	
Primary	320	61 (19.1)	0.33 (0.10–1.07)	0.660
Secondary or higher	197	29 (14.7)	0.24 (0.07–0.81)	0.022
Marital status^*∗*^				
Married/cohabiting	472	84 (17.8)	1	
Single/widow/divorced	56	10 (17.9)	1.0 (0.5–2.1)	0.991
Income category for women^*∗*^				
<60,000 Tsh	355	65 (18.3)	1	
60,000–200,000 Tsh	143	23 (16.1)	0.7 (0.5–1.4)	0.556
>200,001 Tsh	25	5 (20.0)	1.1 (0.4–3.1)	0.833
Gravida				
1st pregnancy	187	34 (18.2)	1	
2nd pregnancy	169	33 (19.5)	1.2 (0.7–2.0)	0.600
≥3rd pregnancy	173	28 (16.2)	1.3 (0.7–2.2)	0.435
Interpregnancy interval				
<24 months	32	8 (25.0)	1	
≥24 months	269	49 (18.2)	0.7 (0.3–1.6)	0.357
Have received iron supplementation at current pregnancy				
No	44	13 (29.5)	1	
Yes	312	63 (20.2)	0.6 (0.3–1.2)	0.160
HIV status at current pregnancy				
Negative	496	90 (18.1)	1	
Positive	32	5 (15.6)	0.8 (0.3–2.2)	0.719
Number of meals taken per day				
1 meal per day	7	3 (42.9)	1	
2 meals per day	29	3 (10.3)	0.2 (0.2–1.0)	0.550
3+ meals per day	315	68 (21.6)	0.4 (0.10–1.7)	0.197
History of food insecurity within past 12 months				
No	342	74 (21.6)	1	
Yes	13	2 (15.4)	0.7 (0.1–3.0)	0.592
Pica habits				
No	135	29 (21.5)	1	
Yes	221	47 (21.3)	1.0 (0.6–1.7)	0.962

^*∗*^Variables with missing information.

**Table 3:** Logistic regression analysis of factors influencing anaemia in pregnancy.

Variable	Adjusted OR (95% CI)	*p* value
*Age group (years)*		
15–24	1	
25–34	0.96 (0.59–1.53)	0.85
35–49	0.42 (0.14–1.24)	0.114
*Education of the woman*		
None	1	
Primary	0.28 (0.08–0.94)	0.04
Secondary or higher	0.21 (0.06–0.74)	0.015
*Name of the clinic enrolled*		
Majengo HC	1	
Pasua HC	2.06 (1.26–3.36)	0.004

**Table 4:** Pregnancy outcomes by anaemia status (*N* = 442).

Variable	*N*	Anaemia (Hb < 11 g/dl) *n* (%)	*p* value
Preterm delivery			
No	440	89 (20.2)	
Yes	2	0 (0.0)	-
Low birth weight (<2500 gms)			
No	426	89 (20.9)	
Yes	16	0 (0.0)	-
Stillbirth			
No	432	79 (18.3)	
Yes	10	1 (10.0)	0.508
Early neonatal death (*N* = 432)			
No	430	79 (18.2)	
Yes	2	0 (0.0)	-
